# Identification of Prognostic Genes and Establishment of a Risk Score Model Related to Pancreatic Adenocarcinoma and Brown Adipose Tissue Based on Transcriptomics and Experimental Validation

**DOI:** 10.3390/genes17010048

**Published:** 2025-12-31

**Authors:** Bin Kang, Weina Wang, Xin Guo, Tong Bai, Chengyu Lv, Yunzhi Shen

**Affiliations:** 1 Department of Hepatobiliary Pancreatic Surgery, Tianjin Third Central Hospital, Tianjin 300170, China; 2 Tianjin Key Laboratory of Extracorporeal Life Support for Critical Diseases, Tianjin 300170, China; 3 Tianjin Artificial Cell Engineering Technology Research Center, Tianjin 300170, China; 4 Tianjin Institute of Hepatobiliary Disease, Tianjin 300170, China; 5 Department of Anesthesiology, Tianjin Hospital, Tianjin 300202, China

**Keywords:** pancreatic adenocarcinoma, brown adipose, prognostic genes, risk score, immune microenvironment

## Abstract

**Background**: Pancreatic adenocarcinoma (PAAD), often referred to as the “king of cancers,” remains poorly understood in terms of the regulatory mechanisms involving brown adipocytes (BAs). **Methods**: Bioinformatics approaches were employed to explore the role of BAs in PAAD progression, utilizing transcriptomic data from public databases. Prognostic genes were identified through differential expression analysis, univariate Cox regression, and machine learning. A risk model categorizing patients into high- and low-risk groups was developed, accompanied by a nomogram. Functional analysis, immune microenvironment profiling, somatic mutation analysis, and drug sensitivity testing were performed, with further validation via gene localization, immunohistochemistry, and clinical sample analysis. **Results**: Six prognostic genes (*SERPINB5*, *CALU*, *TFRC*, *LY6D*, *SFRP1*, and *GBP2*) were identified, with the model and nomogram exhibiting robust predictive performance. Notable differences between the high- and low-risk groups were found in immune pathways, cell infiltration, tumor mutational burden, and drug sensitivity (e.g., axitinib). **Conclusions**: *SERPINB5*, *SFRP1*, and *TFRC* were highly expressed in PAAD samples, providing new insights into potential therapeutic strategies in PAAD treatment.

## 1. Introduction

Pancreatic cancer (PC), a highly malignant tumor primarily arising from the pancreatic ductal epithelium and acinar cells, exhibits a very high degree of malignancy [1]. Both globally and in China, the incidence and mortality of pancreatic adenocarcinoma (PAAD) rank among the top 10 for all malignant tumors, with the 5-year overall survival rate remaining below 10% [2]. Clinical manifestations of PAAD, such as pain and weight loss, are often nonspecific, leading to delayed diagnosis, typically only after the tumor has metastasized to other sites [3]. Current treatment options for PAAD include pancreaticoduodenectomy, pancreatic tail resection with splenectomy, neoadjuvant chemotherapy, combination chemotherapy and radiotherapy, and symptomatic treatment [4,5]. Unfortunately, due to metastasis or invasion of major blood vessels, most patients are not candidates for surgery, and fewer than 20% are eligible for surgical intervention [6]. Existing prognostic models for PC predominantly rely on traditional clinicopathological indicators, which fail to fully capture the tumor’s high heterogeneity, thus limiting prediction accuracy [7]. Moreover, these models generally overlook critical biological factors such as tumor metabolic reprogramming and the immune microenvironment, which are emerging as key drivers of disease progression and determinants of patient outcomes [8]. Consequently, identifying prognostic genes associated with PAAD and developing new prognostic models are critical for offering more personalized treatment options to patients.

Brown adipocytes (BAs) are specialized thermogenic cells rich in mitochondrial uncoupling protein 1 (UCP1) [9]. Upon exposure to cold or pharmacological stimuli, the sympathetic nervous system activates UCP1, causing the proton gradient generated during oxidative phosphorylation to be dissipated as heat instead of being used to generate ATP. This process efficiently consumes glucose and lipids, playing a vital role in thermoregulation and systemic metabolism [10,11,12,13]. Activated BAs may inhibit the growth of various solid tumors by competitively consuming glucose, thus reducing the availability of energy substrates in circulation for tumor utilization [14]. Notably, PAAD is a type of malignancy highly reliant on glycolysis for energy supply. PAAD cells exhibit significant glucose uptake and metabolic reprogramming to sustain rapid proliferation and metastasis. Therefore, systemic competition for energy substrates triggered by BA activation may directly influence the energy supply and survival of PC cells [15]. Additionally, BA function is closely linked to the immune microenvironment [16,17], with its metabolic state potentially modulating the tumor-specific immune response. However, research on the impact of BA-associated genes on PAAD progression and patient prognosis remains limited. This study aims to identify BA-related genes (BARGs) associated with PAAD prognosis and explore their potential biological mechanisms, providing new insights into prognostic assessment and therapeutic strategies for this disease.

Using transcriptomic data from public databases, bioinformatics methods, and experimental validation, this study systematically identified key BARGs significantly associated with PAAD prognosis. The potential value of these prognostic genes and their associated risk models in predicting PAAD outcomes and exploring pathological mechanisms was thoroughly examined through enrichment analysis, immune infiltration analysis, drug sensitivity analysis, and localization and expression analysis. Finally, this study employs bioinformatics methods to explore and screen prognostic genes associated with BA tissues in PAAD and assesses their potential for constructing prognostic models. This provides preliminary clues and candidate targets for understanding the potential association between BA tissues and PAAD and developing novel prognostic tools.

## 2. Methods

### 2.1. Data Acquisition

The clinical and genomic data of patients with PAAD were sourced from The Cancer Genome Atlas (TCGA) database (https://portal.gdc.cancer.gov/, accessed on 13 January 2025) using the TCGAbiolinks package (v 2.30.4) [18] with the keyword “TCGA-PAAD.” The clinical characteristics included age, gender, and disease stage. The TCGA-PAAD dataset consists of 178 tumor tissue samples with complete survival data (PAAD group) and 4 paracancerous normal tissue samples (normal group). This dataset was used for the development of the risk score (RS) model and other related analyses. Additional datasets, GSE71729 (GPL20769), GSE57495 (GPL15048), and GSE155698 (GPL20301), were retrieved from the Gene Expression Omnibus (GEO) database (https://www.ncbi.nlm.nih.gov/geo/, accessed on 13 January 2025). The GSE71729 dataset was used to identify differentially expressed genes (DEGs), while GSE57495 was employed for RS model validation. The GSE71729 dataset contained 145 PAAD tumor tissue samples (PAAD group) and 46 adjacent control tissue samples (normal group), while GSE57495 included 63 PAAD tumor tissue samples (PAAD group). The GSE155698 dataset, used for single-cell analysis, initially contained 15 PC tissue samples and 3 control tissue samples. Two of these PC samples, sequenced using the GPL20301 platform, were excluded due to platform inconsistency. The remaining samples underwent high-throughput single-cell RNA sequencing using the GPL24676 platform. A total of 102 BARGs were retrieved from relevant literature [19] (Table S1). All data were downloaded on 12 February 2025.

### 2.2. Discernment and Related Functional Analysis of Candidate Genes

DEGs were extracted from the GSE71729 dataset using the limma package (v 3.54.0) with thresholds of |log_2_ fold change (FC)| > 0.5 and *p* < 0.05 [20]. A volcano plot of DEGs was generated with the ggplot2 package (v 3.4.1) [21], highlighting the top 10 up-regulated and down-regulated genes. A heatmap of the top 10 DEGs was created using the ComplexHeatmap package (v 2.14.0) [22]. The intersections between DEGs and BARGs were identified using the ggvenn package (v 0.1.9) [23], yielding BARGs in PAAD, designated as candidate genes.

Subsequent analysis of these candidate genes involved Gene Ontology (GO) analysis (*p*.adjust < 0.05) using the clusterProfiler package (v 4.7.1.3) [24]. The GO analysis comprised three major sections: molecular functions (MF), cellular components (CC), and biological processes (BP). The GO terms were ranked by increasing *p*-value, and the five most significant pathways were listed. To explore interactions among the candidate genes, a protein-protein interaction (PPI) network (interaction scores > 0.4) was constructed using the STRING database (https://www.string-db.org, accessed on 14 January 2025). The Cytoscape package (v 3.9.1) [25] was used to visualize the portion of the network with interactions.

### 2.3. Identification of Prognostic Genes

In the TCGA-PAAD dataset, univariate Cox regression analysis was conducted on candidate genes using the survival package (v 3.5.3) [26] to identify genes significantly associated with PAAD survival outcomes (*p* < 0.05, hazard ratio [HR] ≠ 1). Survival-associated genes were subsequently tested for the proportional hazards (PH) assumption (*p* > 0.05) using the cox.zph function from the survival package (v 3.5.3). Genes that satisfied the PH assumption were further analyzed using 10-fold cross-validated LASSO regression with the glmnet package (v 4.1-4) [27]. The LASSO model, with the optimal lambda value, yielded the lowest error rate and identified genes with non-zero regression coefficients as prognostic genes. To assess survival differences among the prognostic genes in PAAD, patients were divided into high-expression and low-expression groups based on the expression levels of each prognostic gene in TCGA-PAAD samples with survival data. Kaplan–Meier (K-M) curves were generated using the survminer package (v 0.4.9) [28], with statistical significance set at *p* < 0.05.

### 2.4. Construction and Evaluation of RS Models

An RS model was then developed using the expression levels of prognostic genes and their respective risk coefficients, as derived from the following formula:

$$\mathrm{RS}=\sum_{i=1}^{10}\beta_{i}*E_{i}$$

In this formula, $\beta_{i}$ represents the regression coefficient for each prognostic gene in the LASSO analysis, and $E_{i}$ represents the gene-specific expression levels. Using the optimal RS threshold, patients in TCGA-PAAD (*n* = 178) and GSE57495 (*n* = 63) with survival data were categorized into the high-risk group (HRG) and the low-risk group (LRG). A risk curve was plotted to illustrate the distribution of RS and survival states across individuals in HRG and LRG. K-M curves for HRG and LRG were generated using the survminer package (v 0.4.9) [28], and survival differences between the groups were assessed using the log-rank test (*p* < 0.05). RS and survival outcome distributions were visualized using the ggplot2 package (v 3.4.1) [21]. Additionally, time-dependent Receiver Operating Characteristic (ROC) analysis (for 1, 2, and 3 years) was performed with the survivalROC package (v 1.0.3.1) [29], and the area under the curve (AUC) was calculated (AUC > 0.6). Finally, the RS model was validated in GSE57495 using the same methods as in TCGA-PAAD to assess its generalizability.

### 2.5. Independent Prognostic Analysis and Construction of Nomogram

Patients with PAAD from TCGA-PAAD were categorized into distinct clinical subgroups based on clinicopathological characteristics, including age, gender, stage, and N/T stage. The Wilcoxon test was then performed to assess significant differences in RS expression levels across these subgroups (*p* < 0.05). Additionally, heatmaps were constructed using the pheatmap package (v 1.0.12) (https://CRAN.R-project.org/package=pheatmap, accessed on 13 January 2025) [22], visually depicting the expression profiles of prognostic genes across distinct RS and clinicopathological subgroups, allowing for comparative analysis of gene expression levels among the different subgroup classifications. Univariate Cox regression analysis identified statistically significant associations (HR ≠ 1, *p* < 0.05), including RS and the aforementioned clinicopathological characteristics. Factors with HR ≠ 1 and *p* < 0.05 were further tested for the PH assumption (*p* > 0.05), and those passing the test were analyzed by multivariate Cox regression (*p* < 0.05, HR ≠ 1) to identify independent prognostic factors. The validity of the multivariate Cox regression results was also assessed by the PH assumption (*p* > 0.05). A nomogram predicting 1-, 2-, and 3-year mortality in patients with PAAD was then developed in TCGA-PAAD using the rms package (v 6.5.0) [30], based on the independent prognostic factors identified above. In the nomogram, each independent prognostic factor was assigned a unique point value, with a higher cumulative score corresponding to a higher risk of PAAD. The nomogram was validated by constructing calibration curves to assess its precision. Additionally, ROC curves for 1-, 2-, and 3-year survival were plotted using the survivalROC package (v 1.0.3.1) [29], and AUC values were calculated to evaluate the predictive performance of the nomogram (AUC > 0.7). Decision curve analysis (DCA) was performed using the rmda package (v 1.6) [31] to further evaluate the clinical relevance of the nomogram.

### 2.6. Function Analyses of HRG and LRG

To explore the biological functional differences between HRG and LRG in TCGA-PAAD, gene set enrichment analysis (GSEA) and gene set variation analysis (GSVA) were performed. DEGs between HRG and LRG were identified using the DESeq2 package (v 1.38.0) [32], and the log_2_FC values were sorted in descending order. The gene set ‘h.all.v2024.Hs.symbols.gmt’ from the Molecular Signatures Database (MSigDB; https://www.gsea-msigdb.org/, accessed on 15 January 2025) was used for GSEA. GSEA was conducted with the cut-offs set at *p* < 0.05, FDR < 0.25, and |NES| > 1, and the top 5 enrichment pathways were visualized using the clusterProfiler package (v 4.7.1.3) in R. Additionally, the ‘c2.cp.kegg.v7.4.symbols.gmt’ gene set from MSigDB was utilized as a reference for GSVA. The GSVA scores for HRG and LRG were analyzed using the single-sample GSEA (ssGSEA) function in the GSVA package (v 1.46.0) [33]. These GSVA scores were compared using the limma package (v 3.54.0) (|t| > 2, *p* < 0.05) [20], and the top 20 enriched pathways were identified.

### 2.7. Immune Microenvironment Analysis

The immune microenvironment between HRG and LRG was compared using the ESTIMATE package (v 1.0.13) [34], with stromal, immune, and ESTIMATE scores calculated and visualized in violin plots. The XCell algorithm [35] was employed to calculate enrichment scores for 64 immune cell types in HRG and LRG samples from TCGA-PAAD. The pheatmap package (v 1.0.12) was used to visualize the results (https://CRAN.R-project.org/package=pheatmap, accessed on 13 January 2025) [22]. Variations in immune cell infiltration across the 64 immune cell types between HRG and LRG were analyzed using the Wilcoxon test, with statistical significance set at *p* < 0.05. Spearman correlation analysis was then performed to explore associations among differential immune cells and between prognostic genes and immune cells using the Spearman function from the psych package (v 2.2.5) [36]. The significance criteria for correlations were set as |correlation coefficient (cor)| > 0.3 and *p* < 0.05.

### 2.8. Somatic Mutation Analysis

Tumor mutation burden (TMB) analysis was conducted on HRG and LRG using tissue samples from patients with PAAD, along with survival data from TCGA-PAAD. This analysis was facilitated by the maftools package (v 2.14.0) [37]. Based on mutation frequencies, the top 20 mutated genes for HRG and LRG were identified, and mutation waterfall diagrams were generated for each group. The Wilcoxon test (*p* < 0.05) was used to compare TMB variations between HRG and LRG.

### 2.9. Drug Sensitivity Analysis

Chemotherapy is a widely used clinical strategy for treating malignant tumors, utilizing drugs with low half-maximal inhibitory concentration (IC50) values to effectively inhibit cell proliferation. A lower IC50 value indicates increased sensitivity of the tumor to the drug, enhancing treatment efficacy. A total of 138 therapeutic agents targeting PAAD were retrieved from the Genomics of Drug Sensitivity in Cancer (GDSC) database (https://ngdc.cncb.ac.cn/databasecommons/database/id/419, accessed on 15 January 2025). IC50 values for these drugs were calculated for each tumor sample from TCGA-PAAD using the pRRophetic package (v 0.5) [38]. The Wilcoxon test (adj *p* < 0.05) was employed to compare differences in IC50 values between HRG and LRG, with the ggplot2 package (v 3.4.1) [21] used to illustrate the top 10 therapeutic agents showing statistically significant differences in IC50 values between the two groups. Additionally, Spearman correlation analysis was conducted between the RS and these 10 drugs, using the Spearman function in the psych package (v 2.2.5) (*p* < 0.05, |cor| > 0.3) [36].

### 2.10. Localization and Immunohistochemistry of Prognostic Genes

To further explore the biology of prognostic genes, chromosome distribution annotation and mapping were performed using the RCircos package (v 1.2.2) [39]. To validate the expression levels of candidate prognostic genes in PAAD tumors and normal pancreatic tissues, immunohistochemical staining images were retrieved from the Human Protein Atlas (HPA; https://www.proteinatlas.org, accessed on 16 January 2025) to compare the differential protein expression patterns encoded by these genes between PAAD and normal tissue samples.

### 2.11. Preprocessing, Dimensionality Reduction, Clustering, and Cell Subpopulation Annotation of Single-Cell Data

To analyze PAAD at the single-cell level, the GSE155698 dataset was first downloaded for high-throughput single-cell sequencing analysis. The Seurat R package (v5.0.1) PMID: 37231261 was then used to filter out low-quality cells, ensuring the accuracy and reliability of subsequent analyses. The filtering criteria were as follows: cells with fewer than 200 expressed genes (nFeature_RNA) were excluded; cells with unique molecular identifier counts (nCount_RNA) below 200 or above 20,000 were removed; cells with mitochondrial gene expression exceeding 15% (percent.mt) were discarded; and cells with nFeature_RNA greater than 7000 were excluded. After quality control, the data were normalized using the vst method to extract genes with high intercellular variability. The FindVariableFeatures function in Seurat (v5.0.1) PMID: 37231261 was applied to select the top 2000 most variable genes, which were then visualized to reduce computational load for further analysis. The ScaleData function from Seurat (v5.0.1) PMID: 37231261 was used to scale the data for these highly variable genes, followed by principal component analysis (PCA) using the JackStrawPlot function. The number of principal components to be used in subsequent analyses was determined based on the significance (*p*-value) of the principal component score plot and the plateau observed in the PCA elbow plot. Next, unsupervised clustering of cells was performed using the FindNeighbors and FindClusters functions from Seurat (v5.0.1), with the clustering results visualized using the UMAP method (resolution = 0.2). Cell clusters were annotated based on clustering results and marker gene information from the literature [40,41] and the CellMarker2.0 website. The annotation was validated visually using the DotPlot function in Seurat (v5.0.1) PMID: 37231261, and cell proportion plots for the Control group, PC group, and the combined group (All) were generated using the ggplot2 package (v3.5.1) PMID: 24132163 to illustrate the distribution characteristics of cell subpopulations across different groups.

### 2.12. Identification of Key Cell Types

To identify key cell types, six previously screened prognostic genes were selected. Bubble plots illustrating the expression of these genes across different cell subpopulations were generated using the ggplot2 package (v3.5.1) PMID: 24132163. Differential expression analysis of the prognostic genes in various cell types was conducted with a significance threshold of *p* < 0.05, thereby identifying key cell types associated with the prognostic genes.

### 2.13. Cell Communication and Pseudotime Analysis

To further investigate the differentiation trajectory, inter-cluster interaction patterns, and core metabolic characteristics of key cells, pseudotime analysis, cell communication analysis, and metabolic activity analysis were performed concurrently. The specific procedures were as follows: sequencing data from the key cells (epithelial cells) were extracted, and a new Seurat object was constructed. Data normalization was performed using the NormalizeData function from Seurat (v5.0.1) PMID: 37231261, with highly variable genes selected using the FindVariableFeatures function and data scaling performed via the ScaleData function. PCA dimensionality reduction and UMAP clustering were then conducted again. Next, the reduceDimension function from the monocle package (v2.26.0) PMID: 37719196, in combination with the DDRTree package (v0.1.5) PMID: 40609536, was used for dimensionality reduction and clustering (parameter setting: max_components = 2). Pseudotime values were assigned to the key cells based on the expression levels of prognostic genes using the orderCells function. Simultaneously, dynamic expression patterns of the prognostic genes along the cell differentiation trajectory were analyzed using the plot_pseudotime_heatmap function. To explore the interactions between cell clusters in the Control and Disease groups, the CellChat package (v1.6.1) PMID: 35592331 was used to analyze cell communication networks among the 16 cell clusters. Visualization methods were employed to present the number, strength, and frequency of interactions within these networks, enabling comparisons of communication patterns between the two groups. Finally, metabolic activity in each subpopulation of the key cells was scored using the AUCell algorithm with the scMetabolism package PMID: 34417225. Heatmaps were generated to visualize the differences in activity across various KEGG metabolic pathways, identifying the core metabolic pathways of the key cells.

### 2.14. The Reverse Transcription Quantitative PCR (RT-qPCR)

To validate the expression of prognostic genes in clinical samples, RT-qPCR analysis was performed. Ten tissue samples (5 PAAD tumors and 5 normal tissues) were collected from the Third Central Hospital of Tianjin. Informed consent was obtained from all participants, and ethical approval was granted by the Third Central Hospital of Tianjin Ethics Committee (IRB2019-034-02). Total RNA was isolated from the samples using TRIzol reagent (Vazyme, Nanjing, China) according to the manufacturer’s protocol. RNA concentration was measured with a NanoPhotometer N50 spectrophotometer. Reverse transcription for cDNA synthesis was performed using Hifair^®^III 1st Strand cDNA Synthesis SuperMix (with gDNA digester plus) on an S1000^TM^ Thermal Cycler (Bio-Rad Laboratories, Inc., Hercules, CA, USA). All primer sequences are listed in Table S2. Quantitative PCR was conducted using a CFX Connect Real-Time PCR Instrument (Bio-Rad, USA), with mRNA relative expression quantified using the 2^−ΔΔCT^ method. RT-qPCR results were analyzed using GraphPad Prism (v 10.1.2) software (https://www.graphpad.com/, accessed on 17 January 2025).

### 2.15. Statistical Analysis

Statistical analysis was performed using R (v 4.2.2). Differences between two groups were assessed using the Wilcoxon test (*p* < 0.05). Statistical significance was indicated as **** (*p* < 0.0001), *** (*p* < 0.001), ** (*p* < 0.01), * (*p* < 0.05), and ns (not significant) (*p* > 0.05).

## 3. Results

### 3.1. Discernment of 25 Candidate Genes and Exploration of Their Biological Functions

**Figure 1:**
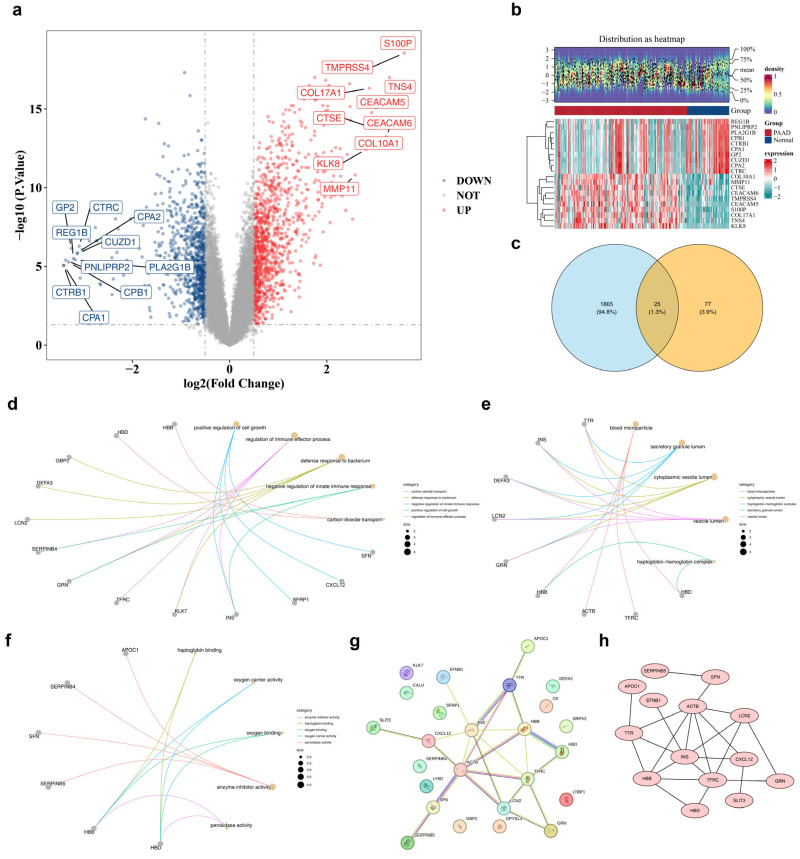
Discernment of candidate genes and exploration of their biological functions (**a**) Volcanic map of differentially expressed genes in PAADvsNormal. The red dots represent the top ten up-regulated genes, while the green dots represent the top ten down-regulated genes. (**b**) Heatmap of differentially expressed genes. Blue: control group; Red: PAAD group. (**c**) Venn diagram of candidate genes revealed 25 candidate genes. (**d**–**f**) BP CC and MF pathway in GO functional enrichment analysis of candidate genes. The annotation of the corresponding molecule and corresponding entry in the connection description indicates that the larger the node, the more molecules the entry contains. (**g**) PPI network construction diagram. Circles represent candidate genes, while lines represent interactions. (**h**) Visualization of Cytoscape in PPI Network. Circles represent candidate genes, while lines represent interactions.

To identify DEGs between the PAAD and normal groups, differential analysis of GSE71729 yielded 1890 DEGs, consisting of 837 down-regulated and 1053 up-regulated genes. These DEGs were visualized in a volcano plot (Figure 1a), with the top 10 most significantly up- or down-regulated genes labeled based on log_2_FC. Heatmaps depicting the expression of these genes were plotted as a proxy for the DEGs (Figure 1b). The intersection of DEGs and BARGs identified 25 candidate genes (Figure 1c and Table S3).

Additionally, 57 significantly enriched GO terms were identified among the candidate genes (*p* < 0.05), including 32 BPs, 13 CCs, and 12 MFs (Table S4). The results revealed that the candidate genes were predominantly enriched in BPs such as “positive regulation of cell growth,” “regulation of immune effector processes,” and “defense response to bacterium” (Figure 1d), while enriched CCs included “blood microparticle,” “secretory granule lumen,” and “cytoplasmic vesicle lumen” (Figure 1e). The main enriched MFs were “haptoglobin binding,” “oxygen carrier activity,” and “oxygen binding” (Figure 1f). Furthermore, 14 candidate genes exhibited protein-level interactions, while 11 candidate genes showed no interactions with other genes (Figure 1g). In the PPI network, there were 14 nodes with 23 pairs of interactions, and *TFRC* interacted with multiple candidate genes (Figure 1h).

### 3.2. Acquisition of 6 Prognostic Genes: SERPINB5, CALU, TFRC, LY6D, SFRP1 and GBP2

**Figure 2:**
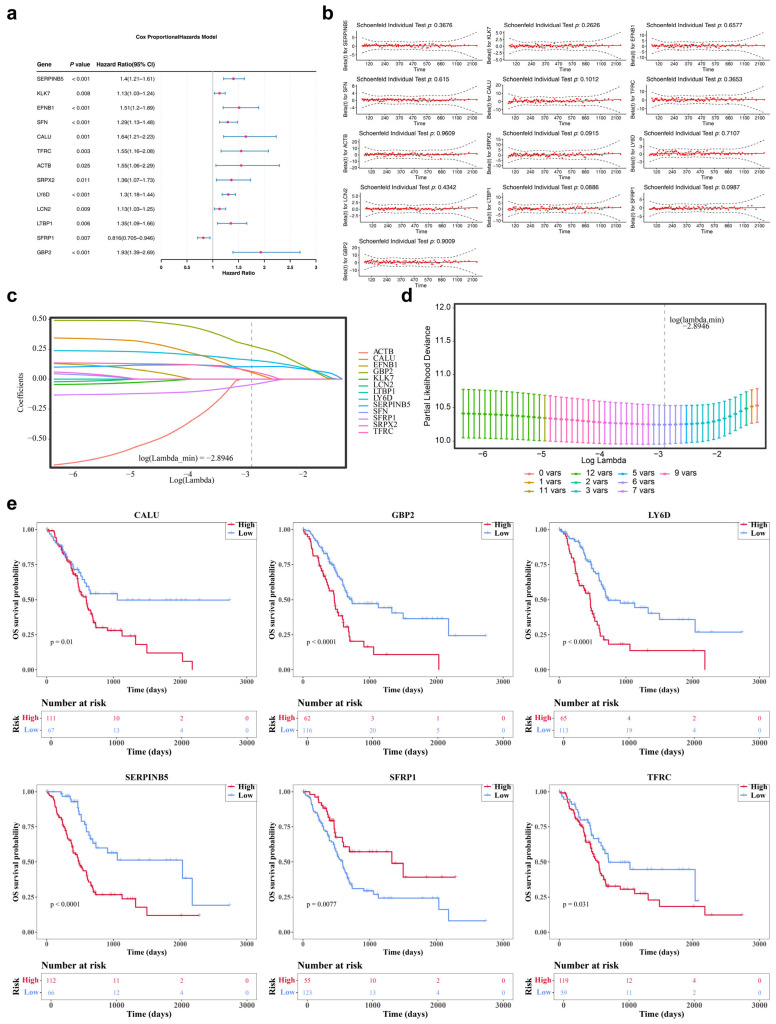
Acquisition of prognostic genes (**a**) Forest plot of single factor COX regression analysis. (**b**) PH hypothesis test. The red dots represent the specific Hazard Ratio values, with HR > 1 indicating risk genes and HR < 1 indicating protective genes. (**c**,**d**) LASSO Regression Algorithm. When reaching the optimal lambda, exclude variables with coefficients equal to 0. The optimal lambda value is at the lowest point of the curve. (**e**) Kaplan–Meier Survival Curves of High/Low Expression Groups for 6 Prognostic Genes (*SERPINB5*, *CALU*, *TFRC*, *LY6D*, *SFRP1*, *GBP2*).

Univariate Cox regression analysis was performed on the 25 candidate genes within the TCGA-PAAD dataset, retaining genes with HR not equal to 1 (*p* < 0.05). The PH assumption was subsequently validated (*p* > 0.05), identifying 13 genes (*SERPINB5*, *KLK7*, *EFNB1*, *SFN*, *CALU*, *TFRC*, *ACTB*, *SRPX2*, *LY6D*, *LCN2*, *LTBP1*, *SFRP1*, and *GBP2*) as significantly associated with survival (Figure 2a,b). Notably, *SFRP1* was identified as a protective factor (*p* = 0.007, HR = 0.816), while high expression of the remaining genes was significantly linked to poorer survival outcomes (*p* < 0.05, HR > 1). The 13 genes were then integrated into the LASSO regression model. According to the LASSO regression analysis, the optimal model was achieved at log(lambda.min) = −2.8946, identifying six candidate prognostic genes (Figure 2c,d). K-M analysis revealed that in TCGA-PAAD, low expression of *SFRP1* (*p* = 0.0077) was associated with a higher survival probability, whereas low expression of *SERPINB5* (*p* < 0.0001), *CALU* (*p* = 0.01), *TFRC* (*p* = 0.031), *LY6D* (*p* < 0.0001), and *GBP2* (*p* < 0.0001) were all linked to lower survival probabilities (Figure 2e). Thus, *SERPINB5*, *CALU*, *TFRC*, *LY6D*, *SFRP1*, and *GBP2* were identified as prognostic genes for PAAD, with low expression of *SFRP1* associated with improved prognosis.

### 3.3. The RS Model Demonstrated a Favorable Predictive Performance

**Figure 3:**
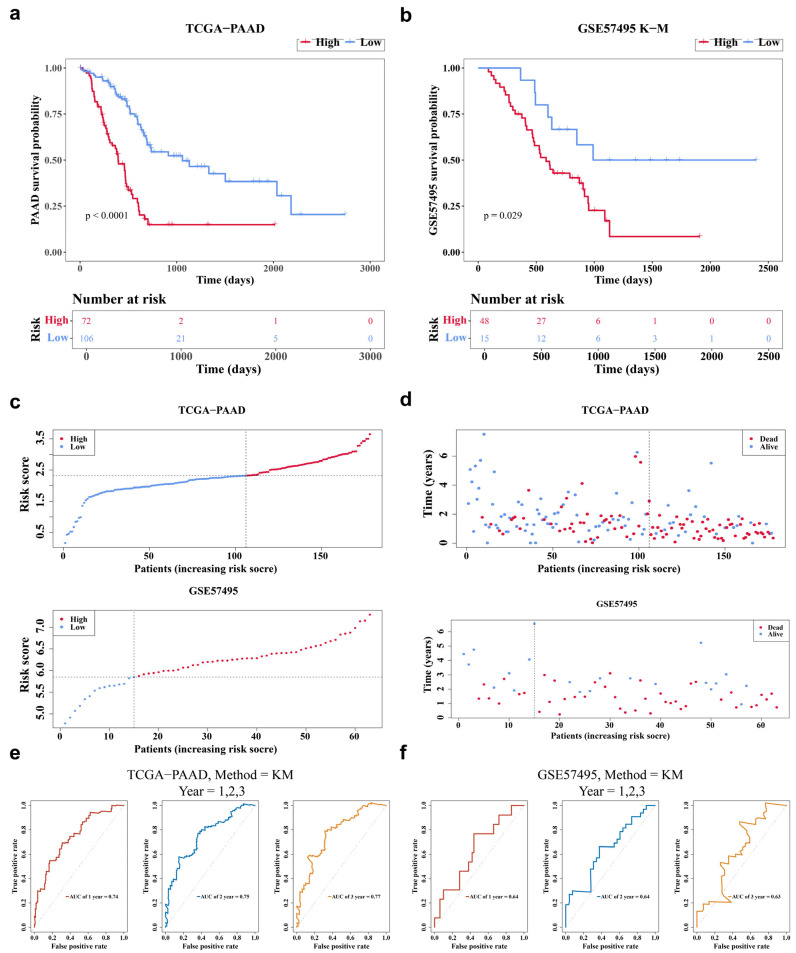
Testing the predictive performance of the RS model. (**a**,**b**) K-M curves of tissue samples from PAAD patients in HRG and LRG. TCGA-PAAD; GSE57495. (**c**,**d**) Risk score curves for patients in HRG and LRG. (**c**): TCGA-PAAD; (**d**): GSE57495. (**e**,**f**) ROC curve analysis of TCGA-PAAD and GSE57495. The larger the AUC, the better the model performance.

An RS model was constructed within the TCGA-PAAD cohort using the prognostic genes *SERPINB5*, *CALU*, *TFRC*, *LY6D*, *SFRP1*, and *GBP2*. The formula for the RS is as follows: RS = (0.10366351) × *SERPINB5* expression + (0.05689634) × *CALU* expression + (0.06567509) × *TFRC* expression + (0.15928227) × *LY6D* expression + (−0.04566806) × *SFRP1* expression + (0.27879245) × *GBP2* expression. Subsequently, 178 PAAD individuals with complete survival data were categorized into HRG (*n* = 72) and LRG (*n* = 106) based on the optimal cut-off value (RS = 2.1343145). In the GSE57495 dataset, 63 tumor samples were divided into HRG (*n* = 48) and LRG (*n* = 15) based on the optimal cut-off value (RS = 5.84789). Survival analysis using K-M curves revealed a significant difference in outcomes between the two cohorts in both TCGA-PAAD (*p* < 0.0001) and GSE57495 (*p* = 0.029), validated by the log-rank test (*p* < 0.05) (Figure 3a,b). As the RS increased, the probability of death correspondingly rose (Figure 3c,d). Furthermore, in TCGA-PAAD, the RS model demonstrated strong predictive accuracy, with AUC values exceeding 0.7 for 1-, 2-, and 3-year survival predictions (Figure 3e). The AUC values in GSE57495 were also greater than 0.6 (Figure 3f), confirming the robust predictability of the RS model for PAAD.

### 3.4. Independent Prognostic Factors: RS and N0/N1 Stage

**Figure 4:**
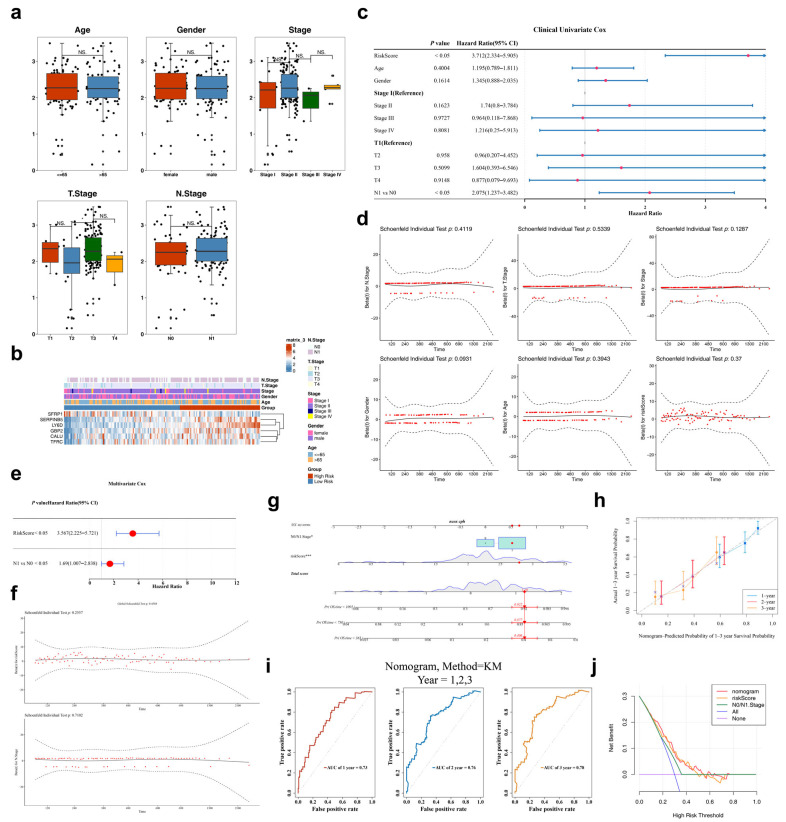
Independent prognostic factors. (**a**) Differences in risk scores among subgroups with different clinical and pathological features (age, gender, pathological stage, TN stage) of PAAD. Ns: *p* ≥ 0.05. (**b**) Heat maps of prognostic gene expression in different risk scores and pathological features. Red: High risk; Blue: Low risk. (**c**,**d**) Univariate Cox regression analysis of PAAD risk score, clinical characteristics, and prognosis. (**e**,**f**) Multivariate Cox regression analysis of the relationship between PAAD risk score, clinical characteristics, and prognosis. (**g**) Construction of Column Chart for Multi factor Independent Prognostic Model. Total points: the total score of a single item representing the value of a variable. * *p* < 0.05, *** *p* < 0.001; (**h**) Calibration curve of column chart model. The actual curve is closer to the ideal curve, indicating that the prediction accuracy of the column chart is higher. (**i**) Column chart model ROC curve. The larger the AUC, the better the model performance. (**j**) Column chart model DCA decision curve. Purple: none; Blue: all; Orange: risk score; Green: N.stage. Red: nomogram.

Subsequent analysis of RS variations across subgroups with distinct clinicopathological characteristics in TCGA-PAAD showed that the difference in RS was statistically significant only between Stage T2 and T3 (*p* < 0.05) (Figure 4a). As presented in Figure 4b, *SERPINB5*, *CALU*, *TFRC*, *LY6D*, and *GBP2* were highly expressed in HRG, while *SFRP1* showed higher expression in LRG. Univariate Cox regression analysis revealed that both RS (HR = 3.712, *p* < 0.05) and N0/N1 stage (HR = 2.075, *p* < 0.05) were significantly associated with survival (Figure 4c,d). Furthermore, multivariate Cox regression indicated that both RS (HR = 3.567, *p* < 0.05) and N0/N1 stage (HR = 1.69, *p* < 0.05) were independent prognostic factors for patients with PAAD (Figure 4e). These factors also passed the PH assumption tests (RS: *p* = 0.2557, N0/N1 stage: *p* = 0.7102) (Figure 4f). To examine the combined prognostic value of RS and N0/N1 nodal status in predicting survival outcomes for PAAD, a nomogram was constructed (Figure 4g). The calibration curve in Figure 4h demonstrates that the actual curve closely approximates the ideal curve, indicating the high prediction accuracy of the nomogram. Additionally, the AUC values for 1-, 2-, and 3-year survival predictions were 0.73, 0.76, and 0.78, respectively (Figure 4i), showing substantial precision in the nomogram’s PAAD predictions. The DCA analysis further validated the favorable clinical applicability of the nomogram (Figure 4j).

### 3.5. Differences in Enrichment Pathways Between HRG and LRG

**Figure 5:**
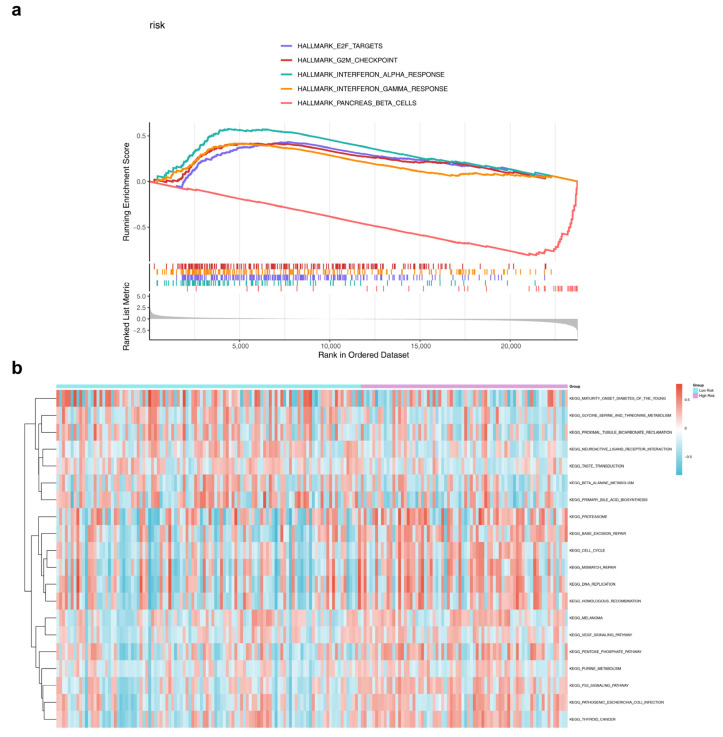
Differences in enrichment pathways between HRG and LRG. (**a**) GSEA diagram of the top 5 pathways in HRG and LRG. The peak value of each line represents the enrichment score of the pathway, and the genes before the peak value are the core genes in the gene set of the pathway. (**b**) Differential analysis of GSVA between HRG and LRG groups. Blue: low enrichment; Red: high enrichment.

The biological pathways involved in HRG and LRG were further examined. GSEA analysis revealed 11 signaling pathways that were significantly enriched in these groups (Table S5). A substantial number of these pathways were associated with immune response processes, such as the “interferon-α response” and “interferon-γ response” (Figure 5a). GSVA identified 186 enriched pathways in HRG and LRG (Table S6). Of these, five pathways showed statistically significant differences between the two groups, including the “p53 signaling pathway” and “base excision repair” (|t| > 2, *p* < 0.05) (Figure 5b).

### 3.6. Estimation of Tumor Immune Microenvironment

**Figure 6:**
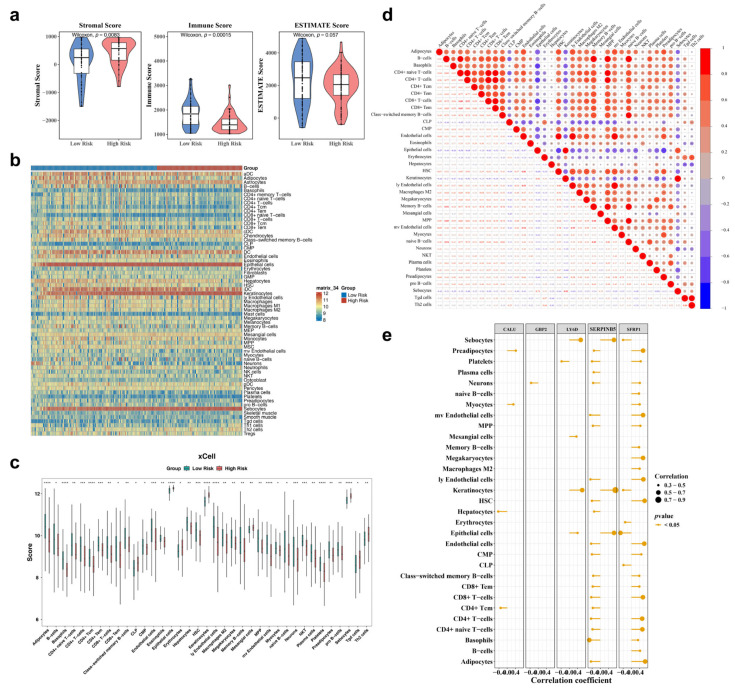
Immune microenvironment of HRG and LRG. (**a**) Analysis of Matrix, Immune, and Estimate Scores in HRG and LRG. Blue: LRG; Red: HRG. (**b**) Distribution map of immune cells in HRG and LRG. The bar chart represents the relative abundance of fractions in different cells. (**c**) Box plot of differential immune cell infiltration abundance between HRG and LRG. *** represented *p* < 0.001, ** represented *p* < 0.01, * represented *p* < 0.05. (**d**) Correlation analysis between differential immune cells. Blue: negative correlation; Red: positive correlation. * *p* < 0.05, ** *p* < 0.01, *** *p* < 0.001; (**e**) Correlation analysis between prognostic genes and differential immune cells. The larger the circle, the higher the correlation.

The immune microenvironment was further analyzed between HRG and LRG. As shown in Figure 6a, there was a significant difference in stromal (*p* = 0.0083) and immune (*p* = 0.00015) scores between the two groups in TCGA-PAAD. However, the ESTIMATE scores (*p* = 0.057) did not show a statistically significant discrepancy between the groups. A heatmap in Figure 6b visualized the enrichment scores of 64 distinct immune cell types in HRG and LRG. Significant differences in immune cell infiltration were observed between the groups in 24 immune cells, including basophils (*p* < 0.0001), and 13 stromal cells, including adipocytes (*p* < 0.0001) (Figure 6c). A strong positive correlation was observed among differential immune cells, with CD4+ and CD8+ T-cells showing a highly significant positive correlation (cor = 0.92, *p* < 0.0001), indicating a prominent co-expression pattern between these lymphocyte populations (Figure 6d). Notably, *SERPINB5* and *SFRP1* exhibited frequent correlations with most differential immune cells. For example, *SERPINB5* showed a strong positive correlation with keratinocytes (cor > 0.7, *p* < 0.05), while *SFRP1* demonstrated a strong negative correlation with epithelial cells (cor < −0.5, *p* < 0.05) (Figure 6e).

### 3.7. Somatic Mutation Analysis Between HRG and LRG

**Figure 7:**
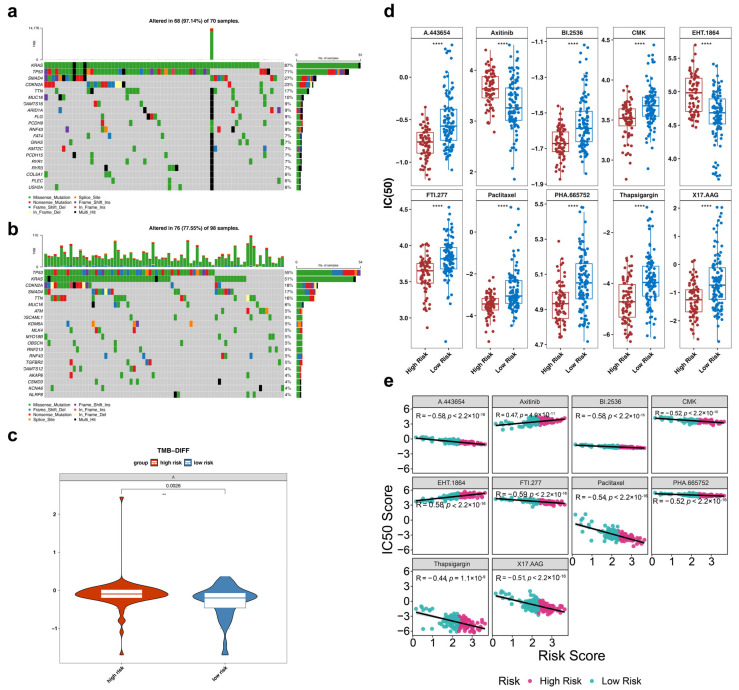
Somatic mutation analysis and Chemotherapy sensitivity analysis between HRG and LRG. (**a**) TMB analysis waterfall plot of HRG. (**b**) TMB analysis waterfall plot of LRG. (**c**) TMB difference between HRG and LRG. **: *p* < 0.01. (**d**) Differences in IC50 of PAAD anti-tumor drugs between HRG and LRG. ****: *p* < 0.0001. (**e**) Correlation analysis between PAAD anti-tumor drugs and risk score. |r| > 0.3 is considered to have a strong correlation, and *p* < 0.05 is considered significant.

Considering the role of genetic alterations in oncogenesis and tumor progression, mutation spectra were derived for patients with PAAD in HRG and LRG from the TCGA database. Somatic variant analysis identified the 20 most commonly mutated genes in both groups. HRG showed the highest percentage of KRAS mutations, with the majority being missense mutations (Figure 7a). In contrast, LRG exhibited the highest percentage of TP53 mutations, also predominantly missense mutations (Figure 7b). A significant difference was observed in the TMB scores between HRG and LRG (*p* = 0.0026) (Figure 7c), with HRG demonstrating higher TMB scores.

### 3.8. Chemotherapy Sensitivity Analysis Between HRG and LRG

A total of 69 chemotherapeutic agents exhibited significant differences in IC50 values between HRG and LRG, as determined by statistical analysis (adjusted *p* < 0.05) (Table S7). Using adjusted *p*-values as the selection criterion, the top 10 drugs showing marked IC50 differences between HRG and LRG were identified: A.443654, Axitinib, BI.2536, CMK, EHT.1864, FTI.277, Paclitaxel, PHA.665752, Trapsigargin, and X17.AAG (Figure 7d). Notably, the IC50 values for these drugs differed significantly between the two groups and were strongly correlated with the RS (|cor| > 0.3, *p* < 0.05) (Figure 7e). For example, FTI.277 showed a pronounced negative correlation with RS (cor = −0.59, *p* < 0.0001).

### 3.9. Quality Control and Annotation of Single-Cell Sequencing Data

**Figure 8:**
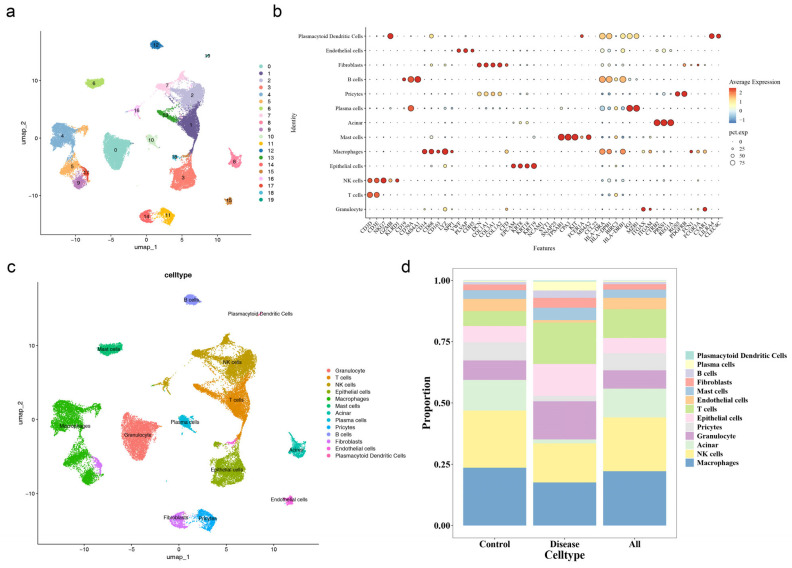
Cell clustering and annotation results. (**a**) UMAP visualization of cell clusters, with different colors representing distinct clusters. (**b**) Visualization of annotated cell clusters. (**c**) Bubble plot showing the expression of marker genes across different cell populations. (**d**) Cell proportion bar plot. The *x*-axis represents the groups: Control, PC, and All (Control + PC). The *y*-axis represents the cell proportion, with different colors corresponding to the various cell types indicated in the legend on the right.

The original single-cell dataset GSE155698 included 49,868 cells and 24,732 genes. After applying a series of filtering criteria for low-quality cells, 35,619 cells met the requirements, with the gene count remaining at 24,732. Post-quality control, the nFeature_RNA, nCount_RNA, and percentage of mitochondrial gene expression (percent.mt) for the cells all fell within acceptable ranges (Figure S1a,b). Following data normalization, 2000 highly variable genes were identified, with *HBB* showing the highest degree of variation. The top 10 highly variable genes were *HBB*, *HBA2*, *CXCL14*, *REG1B*, *HBA1*, *IGJ*, *FXYD2*, *SFRP2*, *PRSS1*, and *PTGDS* (Figure S1c). PCA dimensionality reduction analysis revealed that the *p*-values for the first 50 principal components were all highly significant. Considering the plateau observed in the PCA elbow plot at dimension 30, the first 30 principal components (dims = 30) were selected for further analysis (Figure S1d,e). Using UMAP clustering (resolution = 0.2), all cells were classified into 20 clusters (0–19) (Figure 8a). After annotating these clusters, the 20 clusters were defined as 16 distinct cell types (Figure 8b,c and Table S8). Cell proportion analysis revealed that macrophages and NK cells consistently exhibited the highest proportions in the Control group, the PC group, and the combined group (All) (Figure 8d).

### 3.10. Epithelial Cells as the Key Cell Type

**Figure 9:**
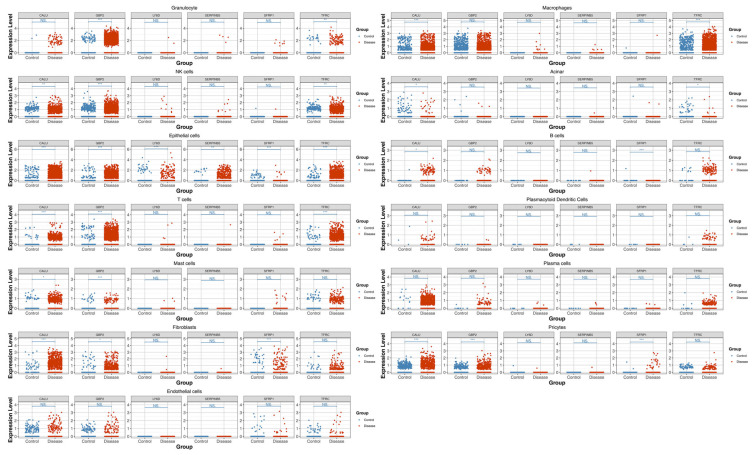
Bubble plot of prognostic gene expression across cells. Blue represents the Control group, and red represents the disease (PC) group. Asterisks denote statistical significance: *, *p* < 0.05; **, *p* < 0.01; ***, *p* < 0.001; NS (no significant) represented *p* > 0.05.

Expression analysis of the six prognostic genes across various cell subpopulations revealed significant differential expression (*p* < 0.05) specifically in epithelial cells, while no significant expression differences were observed in other cell types. Consequently, epithelial cells were identified as the key cell type for subsequent in-depth analysis (Figure 9).

### 3.11. Cell Communication and Pseudotime Analysis

**Figure 10:**
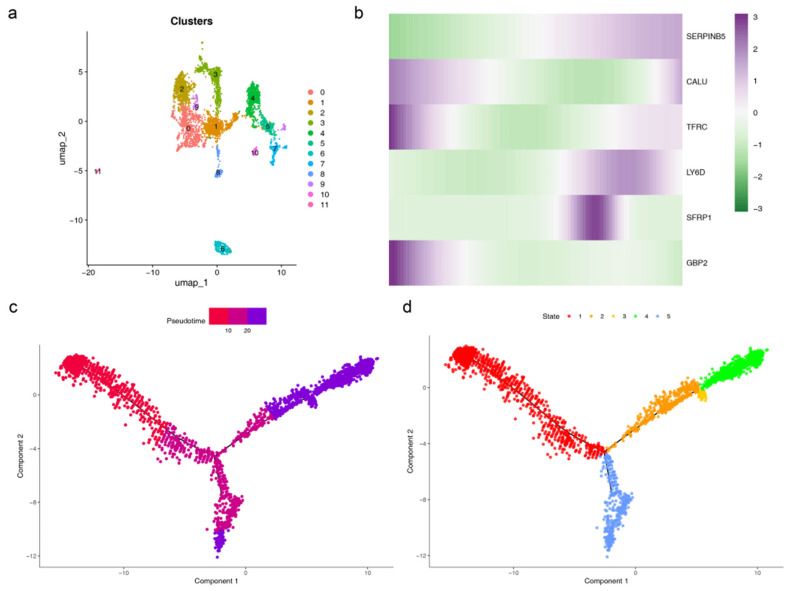
Results of pseudotime trajectory analysis. (**a**) Re-clustered UMAP plot of epithelial cells. (**b**) Heatmap showing the expression of prognostic genes in epithelial cells along the pseudotime axis (from low to high pseudotime, left to right). (**c**) Pseudotime trajectory plot. Labels indicate pseudotime values, with dark red representing the starting point of the developmental trajectory and purple representing the mature state. (**d**) Different developmental stages, with each colored stage representing a distinct cell subpopulation.

**Figure 11:**
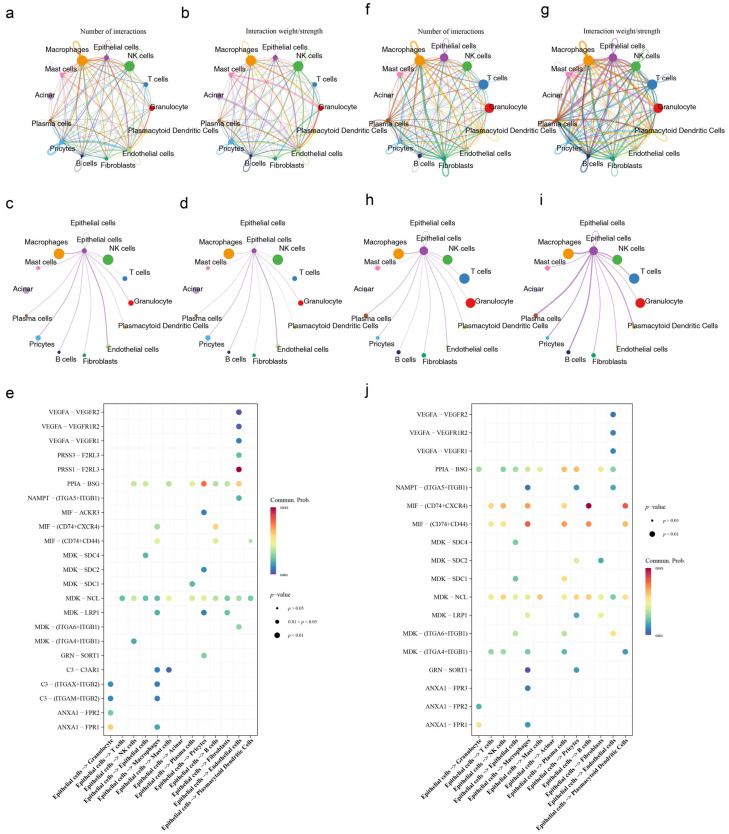
Results of cell-cell communication: (**a**–**e**) Control group, (**f**–**j**) Disease group, (**a**,**f**) number of cell-cell interactions. The size of each colored circle in the outer arc represents the number of cells in that population. Arrows originate from ligand-expressing cells and point to receptor-expressing cells. Thicker lines indicate a greater number of ligand-receptor pairs. (**b**,**g**) Heatmap depicting the strength of cell-cell interactions. (**c**,**h**) Single-network plot showing the number of outgoing signals from key cell populations. (**d**,**i**) Single-network plot showing the strength of outgoing signals from key cell populations. (**e**,**j**) Heatmap analyzing the communication probability between key ligands and their corresponding receptors. The *x*-axis lists ligands (sources), with arrows pointing to receptors (targets). The *y*-axis shows ligand genes and their corresponding receptor genes. The color of the dots represents the communication probability, and the dot size indicates the statistical significance.

In-depth analysis of the key epithelial cells revealed the formation of 12 clusters (0–11) upon re-clustering via UMAP (Figure 10a). The dynamic expression patterns of the prognostic genes exhibited distinct temporal characteristics: *CALU*, *TFRC*, and *GBP2* were highly expressed in the early stages of epithelial cell development; *SERPINB5* and *LY6D* were predominantly expressed in the later stages; and *SFRP1* showed elevated expression during the mid-to-late stages (Figure 10b). The developmental trajectory of epithelial cells was classified into five stages: stage 1 represented the early developmental phase, stages 2, 3, and 5 represented the middle phases, and stage 4 represented the late phase (Figure 10c,d). Cell communication analysis revealed that in the Disease group, communication interactions between epithelial cells and B cells, plasma cells, and plasmacytoid dendritic cells were significantly stronger. Conversely, in the Control group, communication interactions between epithelial cells and endothelial cells were more pronounced, indicating clear differences in core interaction partners between the two groups (Figure 11a–j). Metabolic activity analysis showed that epithelial cells had the highest activity scores in pathways such as fructose and mannose metabolism, ketone body synthesis and degradation, and sulfur metabolism. These results suggest that these metabolic pathways may play a role in regulating epithelial cell function and the progression of PAAD (Figure S2).

### 3.12. Localization Analysis and Clinical Trial Validation of Prognostic Genes

**Figure 12:**
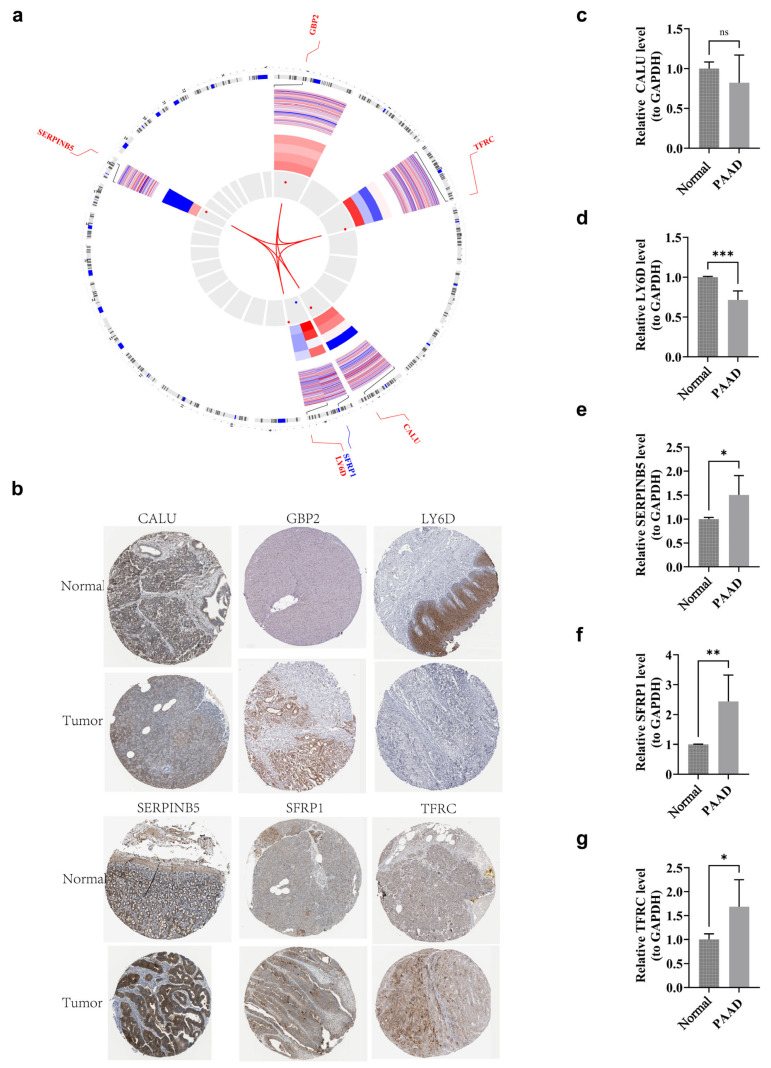
Localization analysis and clinical trial validation of prognostic genes. (**a**) Prognostic gene chromosome localization. Red: up-regulated gene; Blue: down-regulated gene. (**b**) Expression of prognostic genes in PAAD tissues and adjacent control tissues. [Image source: Human Protein Atlas (https://www.proteinatlas.org, accessed on 16 January 2025), used under the CC-BY license.] (**c**–**g**) *CALU*, *LY6D*, *SEPRINB5*, *SFRP1* and *TFRC* level of GAPDH in PAAD tumor samples. *** represented *p* < 0.001, ** represented *p* < 0.01, * represented *p* < 0.05, and ns (no significant) represented *p* > 0.05.

Understanding the chromosomal distribution of genes is valuable for comparative genomics and evolutionary biology studies. Chromosomal localization analysis revealed that *GBP2*, *TFRC*, *CALU*, and *SERPINB5* were clustered on chromosomes 1, 3, 7, and 18, respectively, while *LY6D* and *SFRP1* were localized to chromosome 8 (Figure 12a). The co-localization of *LY6D* and *SFRP1* on chromosome 8 suggests that they may share regulatory elements or belong to a gene cluster, potentially indicating coordinated expression or similar functions. However, further functional studies would be required to confirm this hypothesis. Immunohistochemical staining images of six prognostic gene expression proteins were retrieved from the HPA database. *SERPINB5*, *SFRP1*, and *TFRC* showed elevated expression levels in PAAD tumor samples compared to normal samples. In contrast, the differences in protein expression of *CALU*, *GBP2*, and *LY6D* were less pronounced (Figure 12b). The expression levels of five prognostic genes (*CALU*, *LY6D*, *SERPINB5*, *SFRP1*, and *TFRC*) were further investigated in clinical tissue samples. As shown in Figure 12c–g, *SERPINB5*, *SFRP1*, and *TFRC* exhibited higher expression levels in PAAD tumor samples, consistent with the gene expression data from the HPA database. *LY6D* showed higher expression in normal samples, while *CALU* did not show significant differences between the two groups. These discrepancies could be attributed to various factors, such as post-transcriptional regulation of gene expression, protein stability variations, differences in sample processing, and variations in analytical methods. Therefore, additional studies are necessary to further understand the underlying reasons for these differences.

## 4. Discussion

PC cells heavily rely on glycolysis to fuel their rapid growth and metastasis. In contrast, BA generates heat upon activation by enhancing glucose uptake and lipid metabolism. This process competes for blood glucose, potentially limiting the glycolytic substrates available to tumor cells, thereby inhibiting tumor growth [42]. Against this metabolic backdrop, this study proposes metabolically active BA tissue as a key external factor influencing PAAD progression, exploring its potential prognostic role from a novel metabolic perspective. Through a comprehensive bioinformatics analysis of public datasets, including TCGA, this study successfully identified six PAAD prognostic genes linked to BA function: *SERPINB5*, *CALU*, *TFRC*, *LY6D*, *SFRP1*, and *GBP2*. A PAAD prognostic risk assessment model was constructed based on these genes. Validation results demonstrate that the model exhibits robust predictive performance across both training and validation datasets, with risk scores identified as independent prognostic factors for PAAD. These findings provide new insights into PAAD prognosis. Furthermore, the significant differences in immune microenvironment and mutation patterns between HRG and LRG suggest that the model reflects distinct aspects of tumor biology. This offers valuable guidance for exploring therapeutic strategies targeting metabolic-immune interactions, ultimately enriching our understanding of PAAD progression within a potential metabolic framework. The study also provides fresh perspectives for PAAD treatment and prognostic research.

*SERPINB5*, a BA-associated prognostic gene identified from 102 BARGs in this study, belongs to the cytoplasmic serine protease inhibitor family. Traditionally, *SERPINB5* has been recognized for its tumor-suppressing effects in tissues such as normal mammary epithelium, where it inhibits cell proliferation, invasion, and angiogenesis [43]. However, its function varies markedly across tumor types. Recent studies suggest that *SERPINB5* may have oncogenic properties in several malignancies, including PAAD. For example, its overexpression correlates with poor prognosis in lung adenocarcinoma and promotes tumor invasion in colorectal cancer by activating the TNF-α/NF-κB pathway [44,45]. The experimental data from this study support its potential oncogenic role in PAAD. PCR validation demonstrated that *SERPINB5* is significantly overexpressed in PAAD tissue, with its expression levels positively correlated with the risk model score established here. This suggests that *SERPINB5* may serve as a poor prognostic biomarker for PAAD.

Notably, members of the serine protease inhibitor family can drive endothelial inflammatory responses by enhancing fatty acid oxidation [46]. Chronic inflammatory states are significant negative regulators of BA function, directly impairing the thermogenic activity of BA tissue [47]. Given that BA activation may exert a protective effect on tumors [48], we hypothesize that elevated *SERPINB5* expression in PAAD may indirectly interfere with BA activation and its antitumor function by modulating the local inflammatory environment of the tumor, thus promoting PAAD progression. This hypothesis positions *SERPINB5* as a potential regulatory node linking the malignant phenotype of PAAD with the functional state of BA. Subsequent validation in co-culture systems or in vivo models could directly assess the impact of *SERPINB5* expression on BA activity and PAAD growth, providing theoretical insights and research avenues for PAAD prognosis.

*CALU*, another prognostic gene identified in this study from 102 BARGs, encodes a low-affinity calcium-binding protein primarily localized to the endoplasmic reticulum and Golgi apparatus, and belongs to the *CREC* family [49]. *CALU* plays a critical role in tumor cell survival, invasion, and metastasis by binding to endoplasmic reticulum proteins and regulating endoplasmic reticulum homeostasis. Overexpression of *CALU* has been associated with poor prognosis in lung cancer, colorectal cancer, and breast cancer [50,51]. However, in the PAAD tissues examined in this study, although *CALU* expression showed a downward trend, it did not reach statistical significance. Based on this finding, we speculate that the role of *CALU* in PAAD may differ from that in other tumor types, or that its function in PAAD progression is complex and cannot be fully defined at the transcriptional level. Currently, no definitive literature reports directly linking *CALU* to BA. Notably, BA activation consumes glucose through UCP1-mediated thermogenesis, which may restrict glucose availability in the tumor microenvironment. This could induce metabolic stress in tumor cells, such as endoplasmic reticulum stress [52]. Endoplasmic reticulum homeostasis is essential for the cellular response to such metabolic challenges, and *CALU* serves as a key regulator of endoplasmic reticulum function [53]. Based on these insights and the preliminary findings of this study, we hypothesize that alterations in *CALU* expression may affect PAAD cells’ ability to maintain endoplasmic reticulum homeostasis, potentially modulating the antitumor effects of BA. However, the precise mechanism requires further investigation through subsequent BA activation/inhibition experiments, combined with metabolomics and cellular functional analyses.

*TFRC*, which encodes the protein transferrin receptor 1 (*TFR1*), is a key membrane protein responsible for mediating cellular iron uptake via receptor-mediated endocytosis, playing a critical role in maintaining cellular iron homeostasis [54,55]. Iron is not only an essential cofactor for mitochondrial respiratory chain complexes but is also closely associated with the development and function of BA [56]. Given this, iron homeostasis in adipocytes may play a crucial role in maintaining mitochondrial function and adaptive thermogenesis capacity. Disruption of iron metabolism impairs the thermogenic efficacy of BA [57]. This suggests that *TFR1*-mediated iron uptake may indirectly influence BA activation. Notably, excess iron can generate reactive oxygen species (ROS) through the Fenton reaction, leading to lipid peroxidation and impairing organelle functions, such as those of mitochondria. This can result in iron-dependent cell death, known as ferroptosis [58]. The stability of *TFR1* and its iron uptake process can be regulated by post-translational modifications, such as O-GlcNAc glycosylation, which influence tumor cell susceptibility to ferroptosis [57]. This provides a theoretical foundation for inducing tumor cell death through the regulation of iron metabolism. Given *TFR1*’s role in both tumor iron metabolism and BA-mediated thermogenesis, altered *TFR1* expression may directly support tumor growth by meeting the rapid iron demands of proliferating tumor cells. Concurrently, changes in *TFR1* expression could disrupt intracellular iron homeostasis, potentially affecting both iron-dependent BA thermogenesis and PAAD progression. Future studies simulating BA activation conditions could explore how regulating *TFR1* expression influences PAAD cell sensitivity to ferroptosis and whether this alters tumor response to BA’s antitumor effects, ultimately clarifying its specific role within the BA-PAAD interaction network.

*LY6D*, identified as a BA-associated prognostic gene in this study, encodes a membrane-bound protein primarily involved in cell adhesion pathways. It is highly expressed in various immune cells, including B cells and T cells, suggesting its potential role in the tumor immune microenvironment [59]. In several malignancies, such as laryngeal squamous cell carcinoma, breast cancer, and lung cancer, *LY6D* is associated with tumor progression, metastasis, and poor prognosis. Its mechanisms are thought to involve regulating the miR-509/β-catenin signaling axis and maintaining tumor stem cell properties [60,61,62]. Additionally, *LY6D* is closely linked to drug resistance, migration, and the invasive abilities of tumor stem cells in PAAD [63]. *LY6D* is involved in coordinating multiple immune processes, including T-cell and B-cell activation, dendritic cell maturation, neutrophil and natural killer cell responses, and macrophage polarization [64], suggesting its potential role in shaping the tumor immune microenvironment. This immunoregulatory property is particularly relevant to the anti-tumor mechanism of BA, as the activation and functional state of BA are regulated by the immune system, with immune cell activation correlating with BA activation [65]. Our analysis indicates that risk models based on genes like *LY6D* can distinguish HRG from LRG, with significant differences in immune cell infiltration. Considering this background, *LY6D* may indirectly interfere with immune regulatory networks dependent on or triggered by BA activation, potentially reducing the anti-tumor effects mediated by BA. Further studies are needed to clarify the precise mechanism underlying this interaction.

*SFRP1* encodes a secreted glycoprotein that acts as a classical antagonist of the Wnt/β-catenin signaling pathway. In tumor biology, *SFRP1* is generally recognized for its tumor-suppressive functions, with its silencing or downregulation in PAAD associated with tumor progression and poor prognosis [66,67]. Additionally, *SFRP1* plays a significant role in metabolic regulation. It has been shown to promote the differentiation of precursor cells into BA cells, contributing to BA generation [68]. This study confirmed that the expression levels of *SFRP1* in PAAD tissue are significantly altered. Given its dual role in tumor suppression and adipogenesis, *SFRP1* may not only directly influence Wnt signaling pathway activity in PAAD cells but also indirectly affect BA development or function. Specifically, *SFRP1* downregulation may relieve its inhibitory effect on tumor cell proliferation while diminishing the inherent anti-tumor metabolic capacity of BA tissue, thus promoting PAAD progression. The precise molecular pathways involved in this hypothetical association remain unclear and warrant further investigation to validate *SFRP1*’s actual function in PAAD.

*GBP2* encodes an interferon-induced protein that belongs to the guanine nucleotide-binding protein family [69]. In PAAD, *GBP2* has been shown to directly interact with cyclin proteins such as p27, promoting tumor cell proliferation by regulating the G2/M transition. It is also associated with tumor metastasis and chemotherapy resistance [70]. In addition, *GBP2* exhibits immunomodulatory properties; studies suggest it promotes M1 macrophage polarization in other disease models [71], positioning it as a potential immunotherapy target in colorectal cancer [72]. *GBP2* may also influence the tumor immune microenvironment. Notably, the normal activation and functional maintenance of BA rely on an intact cell cycle process [73]. Therefore, high *GBP2* expression in PAAD may directly support tumor cell proliferation by driving cell cycle progression and may also indirectly modulate the tumor’s response to BA activation by influencing the immune microenvironment or disrupting cell cycle regulation. Future studies should explore and validate this hypothesis.

Building on these findings, a conceptual mechanistic framework has been constructed, as depicted in Figure S3. This framework suggests that these genes collectively attenuate the potential anti-tumor effects of BA activation by regulating the metabolic adaptability of tumor cells, remodeling the local immune microenvironment, and mediating inflammatory signaling pathways. Specifically, *SERPINB5* may modulate the inflammatory environment, while *SFRP1* may interfere with BA function or its generation process. *TFRC* and *CALU* may help tumor cells cope with metabolic stress, such as ferroptosis and endoplasmic reticulum stress. *LY6D* and *GBP2* are primarily involved in regulating immune cells and advancing the tumor cell cycle. These processes are interwoven into a dynamic regulatory network that promotes disease progression, providing a theoretical foundation for future research on the systemic interactions between BA and PAAD.

To further validate the reliability of these findings, this study examined the expression patterns of the six selected BARGs in PAAD. RT-qPCR analysis revealed that *SERPINB5*, *TFRC*, and *SFRP1* showed significantly elevated expression in clinical samples, consistent with protein expression profiles from the HPA database, thereby strengthening their credibility as biomarkers. In contrast, *LY6D* was down-regulated in clinical samples, while *CALU* exhibited a declining trend that did not reach statistical significance. These inconsistencies may be attributed to several factors: complex post-transcriptional regulation, variations in translational efficiency, and differences in protein stability between mRNA transcription levels and final protein abundance [74]. Additionally, differences in tissue origin and pre-processing (such as fixation methods and ischemia time) between the HPA database and the clinical samples in this study may affect the comparability of immunohistochemistry and RT-qPCR results. The inherent heterogeneity of tumors may also contribute to variability in small-sample detection results. Nevertheless, this study demonstrates through multi-cohort cross-validation and prognostic model analysis that the signature constructed based on these genes maintains stable predictive value.

GSVA further explored the relationship between BARGs and PAAD, revealing that the P53 signaling pathway exhibited differential activity across distinct risk groups. As a critical tumor suppressor, P53 plays a central role in tumor initiation and progression by regulating processes such as cell cycle control, DNA repair, apoptosis, and anti-tumor immunity [75]. Elevated intracellular zinc ion levels can inhibit the growth of P53-deficient PAAD cells via ROS/AIF-mediated apoptotic pathways [76], while P53 reactivation-induced autophagy may protect PAAD cells from apoptosis [77]. These findings suggest that BARGs may influence tumor progression through the p53 pathway.

Immunological analysis revealed significant differences in immune and stromal cell infiltration between HRG and LRG, involving 24 immune cell types and 13 stromal cell lineages. Several immune cell subpopulations were significantly correlated with the prognostic gene signature. This points to the importance of the tumor immune microenvironment. Notably, BA tissue itself constitutes an immunologically active environment, housing diverse immune cells such as macrophages, monocytes, dendritic cells, T cells, B cells, natural killer cells, and innate lymphoid cells [17]. These immune cells regulate BA activity through classical pathways, including targeting the sympathetic nervous system. Noradrenaline released from sympathetic nerve endings activates the β-adrenergic receptor-cAMP-PKA pathway, inducing the expression of thermogenic genes like UCP1, while enhancing lipolysis and glucose uptake to support heat production [78]. Consequently, the abundance and activation status of immune cells directly influence the thermogenic efficiency and energy expenditure of BA, impacting metabolic homeostasis by regulating systemic glucose and fatty acid levels [17]. This background provides crucial context for understanding the PAAD immune microenvironment, which is characterized by significant immunosuppression and complexity, playing a pivotal role in tumor growth, invasion, and metastasis [79]. This immunosuppressive environment promotes tumor cell proliferation and survival by suppressing effector T cell function [80]. Based on these findings, BA function may be constrained within the highly immunosuppressed PAAD microenvironment, influencing tumor progression. Future research should further investigate the specific relationship between immune cell infiltration and BA function within a metabolic-immune integration framework, and its broader significance in PAAD progression.

Single-cell transcriptomic sequencing analysis systematically characterized the cellular composition and functional states within the PAAD microenvironment. This analysis identified epithelial cells as the key cell population driving the differential expression of prognostic genes, suggesting their pivotal role in PC progression. Pseudo-temporal trajectories revealed dynamic expression patterns of these genes during epithelial cell development. Notably, *CALU*, *TFRC*, and *GBP2* were highly expressed in early developmental stages, whereas *SERPINB5* and *LY6D* were up-regulated in later phases, indicating their involvement in distinct stages of cellular state transitions and tumor progression. Cellular communication analysis showed significantly enhanced interactions between epithelial cells and B cells, plasma cells, and plasma cell-like dendritic cells in disease conditions. This suggests that reconfigured communication between epithelial cells and immune cells within the tumor microenvironment may influence immune regulation and disease progression. Metabolic activity assessments revealed increased epithelial cell activity in pathways such as fructose and mannose metabolism, ketone body synthesis and breakdown, and sulfur metabolism, reflecting metabolic reprogramming in response to tumor-related metabolic stress. These findings provide valuable insights at the single-cell level into cellular heterogeneity, gene expression dynamics, and intercellular interactions within the PC microenvironment. However, these discoveries are based primarily on bioinformatics correlation analyses, and their underlying biological mechanisms require further validation through subsequent experimental studies.

Notably, the PAAD risk model developed in this study, based on prognostically relevant genes associated with BA, and its integrated prognostic nomogram combining clinical and pathological parameters, demonstrates potential clinical utility. This model enables differentiation between HRG and LRG patients through multi-gene expression signatures, with the risk score serving as a supplementary prognostic factor alongside conventional clinical indicators. By integrating gene scores with clinical characteristics, the nomogram offers a practical tool for visualizing patient survival probabilities, providing a reference for subsequent individualized treatment strategies. However, this study is still in the early validation stage, and the clinical applicability of the model requires further confirmation through systematic translational research. Future work should focus on several priorities: First, developing the six-gene biomarker into a standardized diagnostic kit to facilitate clinical implementation and risk stratification; second, organizing multicenter, prospective cohort studies to expand sample sizes and validate the model’s robustness and generalizability, as well as exploring its integration with imaging and serological indicators; third, designing differentiated management pathways based on risk stratification, such as intensifying treatment and follow-up for HRG patients while avoiding excessive intervention for LRG patients; and fourth, further investigating the interaction mechanisms between BA-associated genes and tumor metabolism/immune microenvironment to explore targeted combination therapies for HRG patients. Through these steps, the model can be progressively translated into clinical practice, ultimately improving the accuracy of individualized prognosis assessment and treatment decisions for patients with PAAD.

Finally, it is important to emphasize that this study primarily relies on bioinformatics analysis and correlation validation using clinical samples. The specific biological functions of these genes in PAAD and their direct causal relationship with BA tissue activity remain unconfirmed. The current findings represent preliminary, hypothesis-driven research, and future experimental validation is required to explore the causal link between BA tissue and PAAD.

## 5. Conclusions

This study identified six BA-related prognostic genes (*SERPINB5*, *CALU*, *TFRC*, *LY6D*, *SFRP1*, *GBP2*) in PAAD through systematic bioinformatics screening and developed a risk assessment model based on these markers, offering a new molecular framework for PAAD prognosis. However, several limitations exist: First, functional validation is insufficient, with findings primarily based on bioinformatics and clinical tissue expression data, lacking in vitro (e.g., gene modification in PAAD cells) and in vivo (e.g., PAAD xenografts with BA regulation) experiments to confirm gene roles; second, the model has limited generalizability, as it was built and validated using a small dataset and local samples without multi-center external verification; third, the reasons for discrepancies between the HPA database and clinical tissue expression (e.g., post-transcriptional regulation, protein stability) remain unaddressed. To overcome these challenges, future work will focus on enhancing functional validation (in vitro: gene-modified PAAD cells under BA co-culture; in vivo: nude mouse PAAD models with BA regulators), optimizing the model (expanding multi-center samples, integrating clinical parameters, and conducting external validation), investigating expression discrepancies (RNA sequencing, proteomics, and molecular interaction verification), and facilitating clinical translation (developing rapid gene detection kits and designing targeted therapies, such as high-risk gene silencing combined with BA activation).

## Data Availability

The datasets [TCGA-PAAD, GSE71729, GSE57495 and GSE155698] for this study can be found in The Cancer Genome Atlas (TCGA) [https://portal.gdc.cancer.gov/, accessed on 13 January 2025] and [GEO] [https://www.ncbi.nlm.nih.gov/geo/, accessed on 13 January 2025]. The original contributions presented in this study are included in the article/Supplementary Materials. Further inquiries can be directed to the corresponding author.

## Supplementary Materials

The following supporting information can be downloaded at: https://www.mdpi.com/article/10.3390/genes17010048/s1, Figure S1. Quality control results of the single-cell RNA sequencing data. (a,b) nFeature_RNA (number of genes detected per cell), nCount_RNA (total number of molecules detected per cell), and percent.mt (percentage of mitochondrial genes per cell) (a) before and (b) after quality control. (c) Screening of highly variable genes. Genes shown in red represent the top 2000 highly variable genes, while those in black are non-highly variable genes. (d,e) Principal component analysis: (d) Scree plot of principal components. (e) Scatter plot of principal components. Figure S2. Heatmap of cell metabolic pathway activity. Both the size and the color intensity of the circles represent the metabolic activity score; Figure S3. Mechanism Hypothesis Diagram. Table S1. List of 102 brown adipocyte-related genes (BARGs); Table S2. All primer sequences are listed; Table S3. Detailed information of candidate genes; Table S4. GO enrichment analysis results of candidate genes; Table S5. GSEA enrichment pathway results of HRG and LRG; Table S6. GSVA enrichment pathway results of HRG and LRG; Table S7. IC50 differences in chemotherapeutic agents between HRG and LRG; Table S8. Cell annotation Marker gene table.

## Abbreviations

The following abbreviations are used in this manuscript:

Abbreviation	Full Form
PAAD	Pancreatic Adenocarcinoma
BA	Brown Adipocyte
PH	Proportional Hazards
DCA	Decision Curve Analysis
RS	Risk Score
UCP1	Uncoupling Protein 1
TCGA	The Cancer Genome Atlas
GEO	Gene Expression Omnibus
BARGs	Brown Adipocyte-Related Genes
DEGs	Differentially Expressed Genes
GO	Gene Ontology
MF	Molecular Functions
CC	Cellular Components
BP	Biological Processes
PPI	Protein-Protein Interaction
HR	Hazard Ratio
K-M	Kaplan-Meier
HRG	High Risk Group
LRG	Low Risk Group
ROC	Receiver Operating Characteristic
AUC	Area Under Curve
GSEA	Gene Set Enrichment Analysis
GSVA	Gene Set Variation Analysis
MSigDB	Molecular Signatures Database
ssGSEA	Single Sample GSEA
TMB	Tumor Mutation Burden
IC50	Half-Maximal Inhibitory Concentration
GDSC	Genomics of Drug Sensitivity in Cancer
HPA	Human Protein Atlas
RT-qPCR	Reverse Transcription Quantitative PCR
