# Supplementary material for: Identification of Prognostic Genes and Establishment of a Risk Score Model Related to Pancreatic Adenocarcinoma and Brown Adipose Tissue Based on Transcriptomics and Experimental Validation
Source: Genes (Basel). 2025 Dec 31;17(1):48. doi: 10.3390/genes17010048 (PMC12840907; doi:10.3390/genes17010048)
Supplement: Supplementary file 1 [file genes-17-00048-s001.zip › Supplementary Tables/Table S3 .pdf]

Table S3 Detailed information of candidate genes

Gene	logFC	P.Value	adj.P.Val	change
ACTB	0.713920689655174	3.25540373075387e-06	4.09757605345176e-05	UP
APOC1	0.624113793103447	0.00473784104512788	0.0162443789583733	UP
C6	-1.502485007	1.90820380693901e-06	2.6170220127249e-05	DOWN
CALU	0.63115652173913	1.10629892528511e-09	5.84179076883839e-08	UP
CXCL12	-1.200164918	2.09107571435028e-07	4.21824864991865e-06	DOWN
DEFA3	-1.023831334	0.00029459865022647	0.00167908477440768	DOWN
DPYSL3	0.617199400299848	0.00043720462777676	0.00231174141739313	UP
EFNB1	1.14371364317841	1.85029521572379e-10	1.30040854858822e-08	UP
GBP2	0.532371064467767	0.00085468146198889	0.00402938748933367	UP
GRN	0.698538380809596	1.1673015031808e-06	1.71781202580608e-05	UP
HBB	-1.661473913	1.20946945981492e-05	0.00012124777848672	DOWN
HBD	-1.222377661	1.15989736395311e-10	8.84432935934751e-09	DOWN
INS	-1.642012744	0.00661264162285112	0.0212796251278616	DOWN
KLK7	2.18006476761619	2.87176644628662e-13	6.75172804139457e-11	UP
LCN2	1.4466035982009	9.04653699454334e-05	0.00063479101982238	UP
LTBP1	0.606502848575713	0.000175249665979	0.00108325685553031	UP
LY6D	1.42781184407796	2.95270663430951e-05	0.00025254657133381	UP
SERPINB4	1.35863133433283	1.96995626468225e-05	0.00018036470223092	UP
SERPINB5	1.21627031484258	1.55558927887342e-14	6.42450645411575e-12	UP
SFN	0.976599550224887	5.2905894312904e-10	3.15661180297747e-08	UP
SFRP1	-0.7056991	0.00084408509361505	0.00398799916598175	DOWN
SLIT3	-0.703068666	1.25057569421569e-06	1.81867594882663e-05	DOWN
SRPX2	0.808379910044975	1.94123426608985e-05	0.0001781694982188	UP
TFRC	0.724971814092955	1.79918663978797e-07	3.70512376946535e-06	UP
TTR	-1.225742729	0.0157348768851379	0.0424345327877357	DOWN
