# Supplementary material for: Identification of Prognostic Genes and Establishment of a Risk Score Model Related to Pancreatic Adenocarcinoma and Brown Adipose Tissue Based on Transcriptomics and Experimental Validation
Source: Genes (Basel). 2025 Dec 31;17(1):48. doi: 10.3390/genes17010048 (PMC12840907; doi:10.3390/genes17010048)
Supplement: Supplementary file 1 [file genes-17-00048-s001.zip › Supplementary Tables/Table S8.pdf]

Table S8 Cell annotation Marker gene table

Marker gene	Cell
CD3D, CD3E	T cells
NKG7, GZMB, KLRD1	NK cells
CD19, CD79A, MS4A1	B cells
CD14, CD68, CD163, LYZ, SPP1	Macrophages
VWF, PLVAP, CDH5	Endothelial cells
DCN, COL1A1, COL3A1, COL1A2, CFD	Fibroblasts
EPCAM, KRT8, KRT18, KRT19	Epithelial cells
NCAM1, SYT1, SNAP25	Neural cells
TPSAB1, CPA3, KIT, FCER1A, MS4A2	Mast cells
CCL22, HLA-DRA, HLA-DPB1, BIRC3, HLA-DRB1	Dendritic cells
IGJ, CD79A, MZB1	Plasma cells
ITGAX, ITGAM	Granulocyte
CTRB2, PRSS1, REG1A	Acinar
RGS5, PDGFRB	Pericytes
FCN1, FCGR3A, LYZ, C5AR1	CD16+ Monocyte
LILRA4, CLEC4C	Plasmacytoid Dendritic Cells
