# Supplementary figures and images for: Identification of Prognostic Genes and Establishment of a Risk Score Model Related to Pancreatic Adenocarcinoma and Brown Adipose Tissue Based on Transcriptomics and Experimental Validation
Source: Genes (Basel). 2025 Dec 31;17(1):48. doi: 10.3390/genes17010048 (PMC12840907; doi:10.3390/genes17010048)

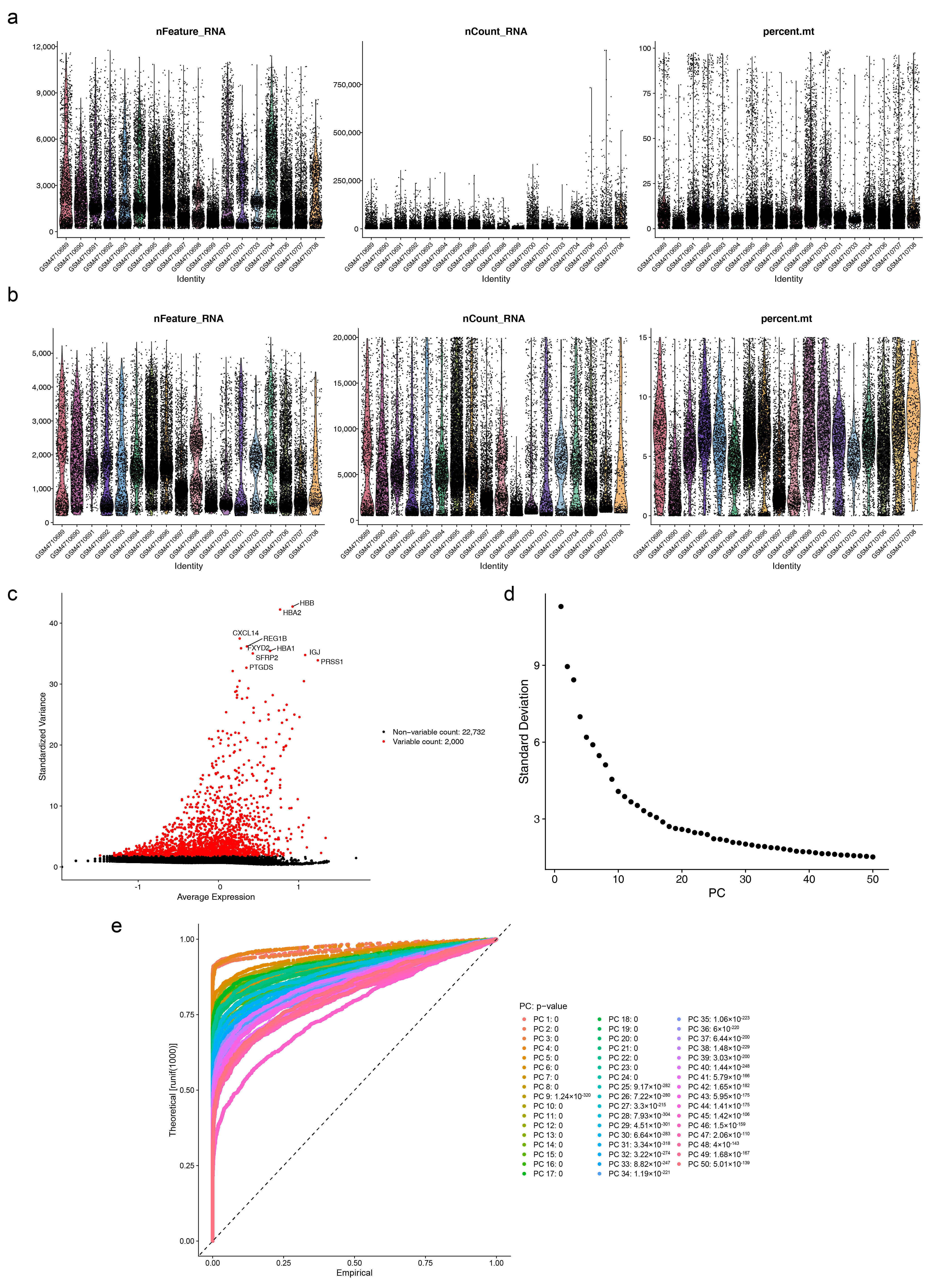

Supplement: Supplementary file 1 [file genes-17-00048-s001.zip › Supplementary Figures/Figure S1.tif]

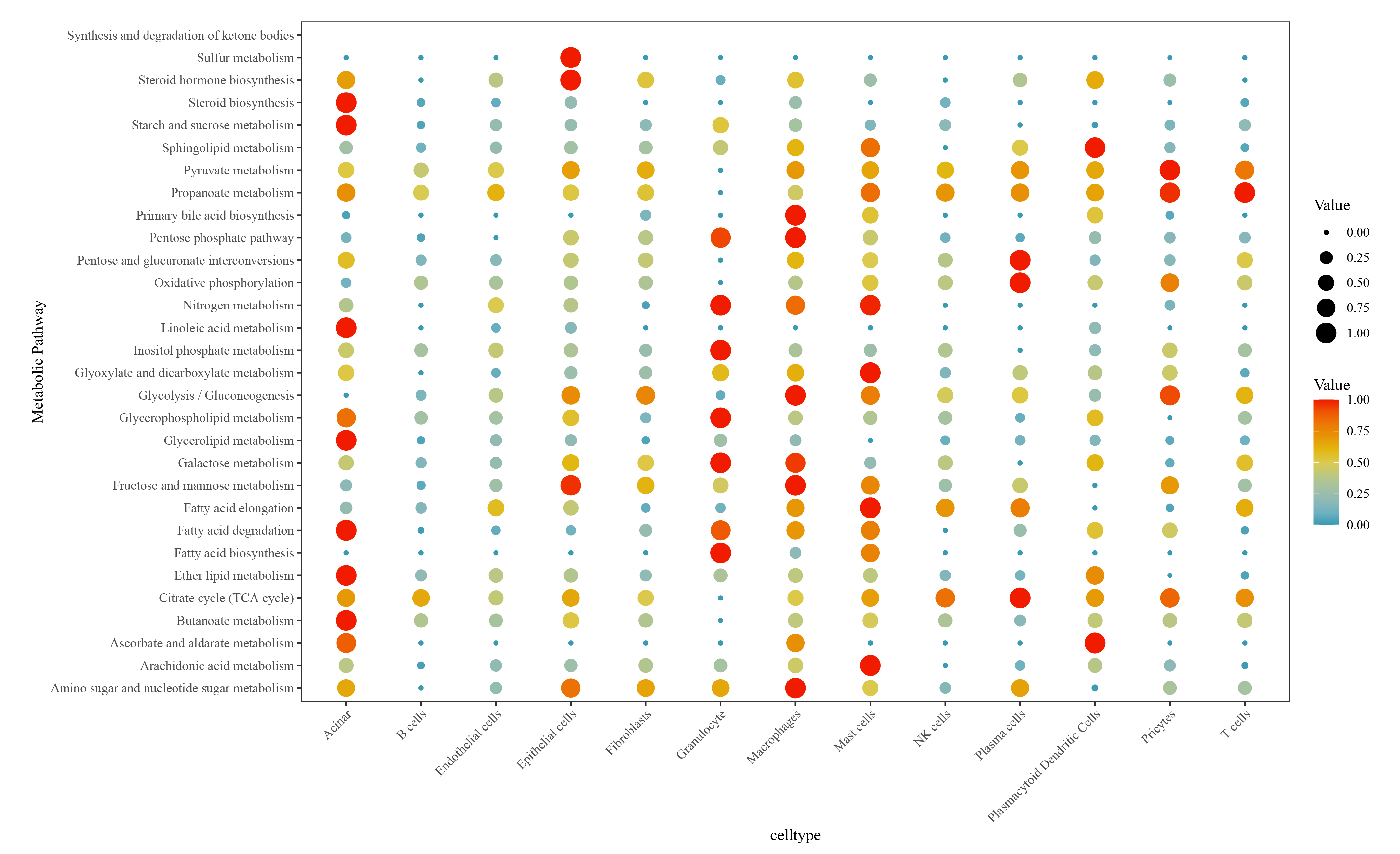

Supplement: Supplementary file 1 [file genes-17-00048-s001.zip › Supplementary Figures/Figure S2.tif]

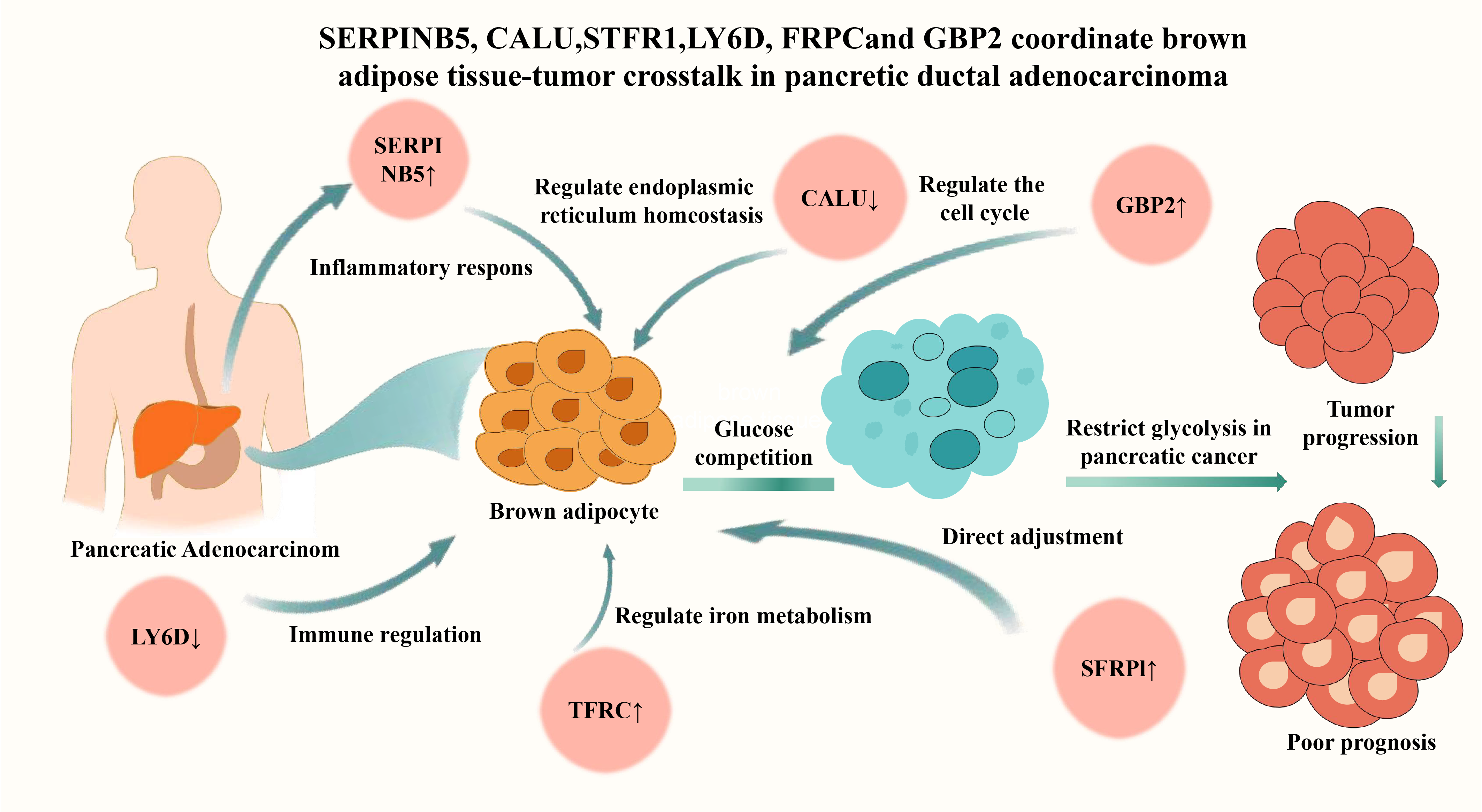

Supplement: Supplementary file 1 [file genes-17-00048-s001.zip › Supplementary Figures/Figure S3.tif]
